# Biflavans, Flavonoids, and a Dihydrochalcone from the Stem Wood of *Muntingia calabura* and Their Inhibitory Activities on Neutrophil Pro-Inflammatory Responses

**DOI:** 10.3390/molecules191220521

**Published:** 2014-12-08

**Authors:** Wen-Lung Kuo, Hsiang-Ruei Liao, Jih-Jung Chen

**Affiliations:** 1 Chung-Jen Junior College of Nursing, Health Sciences and Management, Chiayi 600, Taiwan; 2 Graduate Institute of Natural Products, College of Medicine, Chang Gung University, Taoyuan 333, Taiwan; E-Mail: liaoch@mail.cgu.edu.tw; 3 Department of Pharmacy, Tajen University, Pingtung 907, Taiwan; 4 Graduate Institute of Pharmaceutical Technology, Tajen University, Pingtung 907, Taiwan

**Keywords:** *Muntingia calabura*, Tiliaceae, biflavan, flavone, dihydrochalcone, structure elucidation, anti-inflammatory activity

## Abstract

*Muntingia calabura* (Tiliaceae) is commercially used in healthcare for the improvement of hypertension, myocardial infarction, spasm, and inflammatory conditions. Its fruits can be processed into jam and the leaves can be used for making tea. In the work reported herein a new biflavan, (*M*),(2*S*),(2''*S*)-,(*P*),(2*S*),(2''*S*)-7,8,3',4',5',7'',8'',3''',4''',5'''-decamethoxy-5,5'' biflavan (**1**), a new flavone, 4'-hydroxy-7,8,3',5'-tetramethoxyflavone (**2**), and a new dihydrochalcone, (*R*)-2',β-dihydroxy-3',4'-dimethoxydihydrochalcone (**3**), have been isolated from the stem wood of *M. calabura*, together with 12 known compounds (**4**–**15**). The structures of these new compounds were elucidated by the interpretations of extensive spectroscopic data. Among the isolated compounds, 5-hydroxy-7-methoxyflavone (**5**), quercetin (**6**), and (2*S*)-7-hydroxyflavanone (**10**) exhibited potent inhibition of fMLP-induced superoxide anion generation by human neutrophils, with IC_50_ values of 1.77 ± 0.70, 3.82 ± 0.46, and 4.92 ± 1.71 μM, respectively.

## 1. Introduction

*Muntingia calabura* L. (Tiliaceae) is an evergreen tree originally distributed in tropical America [1]. In Mexico, the fruits of *M. calabura* are eaten and sold in markets. The fruits can be processed into jams and the leaves can be used for making tea. Past studies have revealed flavones, flavanones, flavans, and biflavans to be the major constituents of this species, some of which have displayed anti-platelet aggregation [2] and cytotoxic [3,4,5,6] activities.

**Figure 1:**
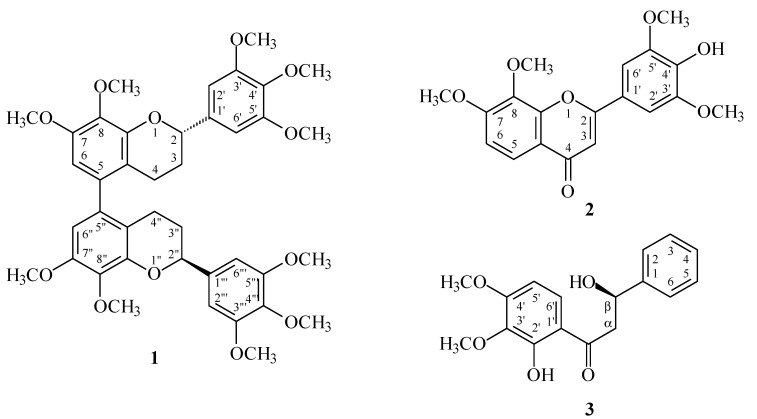
The chemical structures of new compounds **1**–**3** isolated from *M. calabura*.

**Figure 2:**
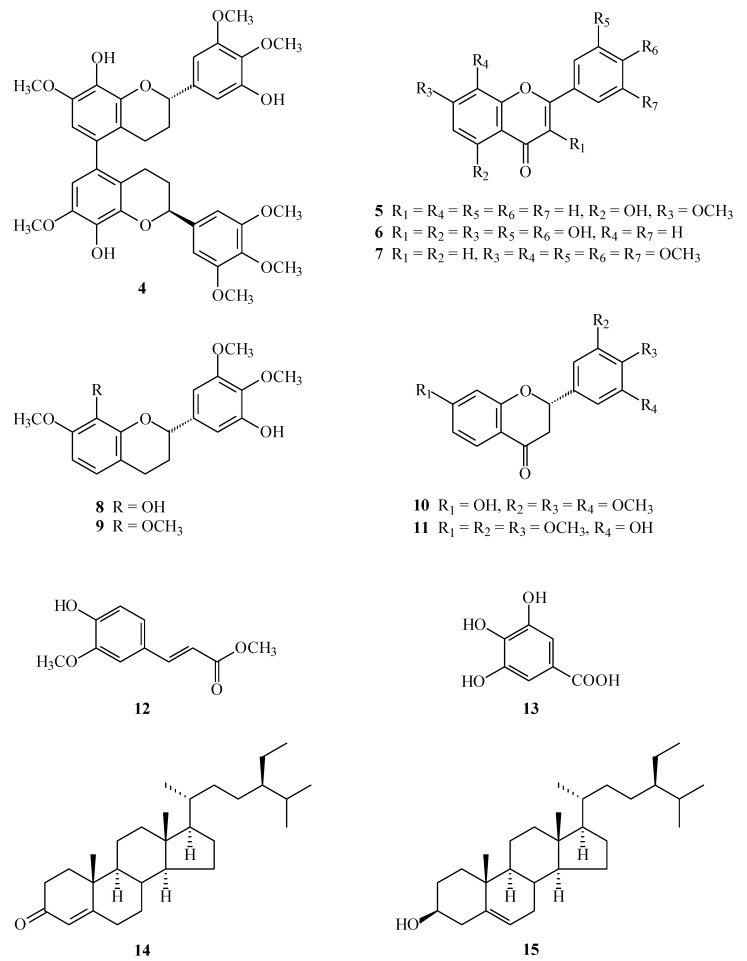
The chemical structures of known compounds **4**–**15** isolated from *M. calabura*.

Reactive oxygen species (ROS) (e.g., superoxide anion (O_2_^•−^) and hydrogen peroxide) produced by human neutrophils are involved in the pathogenesis of a variety of inflammatory diseases. In a screening program searching for anti-inflammatory compounds from Formosan plants [7,8,9,10,11], *M. calabura* was found to be an active species. The MeOH extract of the stem wood of *M. calabura* showed potent inhibitory effects on superoxide anion generation by human neutrophils in response to formyl-l-methionyl-l-leucyl-l-phenylalanine (fMLP). Figure 1 shows the structures of three new compounds, (*M*),(2*S*),(2''*S*)-,(*P*),(2*S*),(2''*S*)-7,8,3',4',5',7'',8'',3''',4''',5'''-decamethoxy-5,5''-biflavan (**1**), 4'-hydroxy-7,8,3',5'-tetramethoxyflavone (**2**), and (*R*)-2',β-dihydroxy-3',4'-dimethoxydihydrochalcone (**3**) that have been isolated and identified from the stem wood of *M. calabura* together with the 12 known compounds **4**–**15** (Figure 2). This paper describes the structural elucidation of the compounds **1**–**3**, and the inhibitory activities of all isolates on superoxide generation by neutrophils.

## 2. Results and Discussion

### 2.1. Results

**Figure 3:**
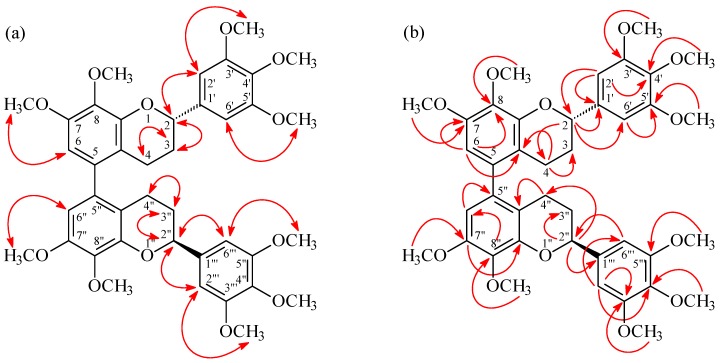
Key NOESY (**a**) and HMBC (**b**) correlations of **1**.

Chromatographic purification of the CH_2_Cl_2_-soluble fraction of a MeOH extract of stem wood of *M. calabura*on a silica gel column and preparative thin-layer chromatography (TLC) afforded three new compounds **1**–**3** and twelve known compounds **4**–**15**.

(*M*),(2*S*),(2''*S*)-,(*P*),(2*S*),(2''*S*)-7,8,3',4',5',7'',8'',3''',4''',5'''-Decamethoxy-5,5''-biflavan (**1**) was obtained as optically active (${\left[\alpha \right]}_{D}^{25}$ = +12.8) colorless prisms. The molecular formula C_40_H_46_O_12_ was deduced from a sodium adduct ion at *m*/*z* 741.2889 [M + Na]^+^ (calcd. 741.2887) in the HR-ESI mass spectrum. The presence of an aromatic ring C=C stretch was revealed by the bands at 1602, 1515, and 1452 cm^−1^ in the IR spectrum. The ^1^H-NMR spectrum (Table 1) of **1** indicated the presence of five methoxy groups [δ 3.85 (3H, s, OMe-4'), 3.86 (3H, s, OMe-7), 3.87 (6H, s, OMe-3' and OMe-5'), 3.90 (3H, s, OMe-8)], three aromatic protons [δ 6.42 (1H, s, H-6), 6.64 (2H, s, H-2' and H-6' )], an oxymethine proton [δ 5.02 (1H, dd, *J* = 9.6, 3.0 Hz, H-2)], and four methylene protons [δ 1.90–2.08 (1H, m, H_ax_-3), 2.09-2.18 (1H, m, H_eq_-3), 2.23 (1H, ddd, *J* = 16.4, 4.4, 4.4 Hz, H_eq_-4), 2.66 (1H, ddd, *J* = 16.4, 11.0, 6.0 Hz, H_ax_-4)]. Comparison of the ^1^H-, ^13^C-NMR, and MS data of **1** with those of (*M*),(2*S*),(2''*S*)-, (*P*),(2*S*),(2''*S*)-8,5',8''-trihydroxy-7,3',4',7'',3''',4''',5'''-heptamethoxy-5,5''-biflavan (**4**) [5] (Table 1) suggested that their structures were closely related, except that the 8-methoxy [δ_H_ 3.90 (3H, s); δ_C_ 60.8 (OMe-8)], 5'-methoxy [δ_H_ 3.87 (3H, s); δ_C_ 56.1 (OMe-5')], and 8′′-methoxy [δ_H_ 3.90 (3H, s); δ_C_ 60.8 (OMe-8'')] groups of **1** replaced the 8,5',8''-trihydroxy groups of **4** [5]. This was supported by both HMBC correlations (Figure 3) between OMe-8 (δ 3.90)/C-8 (δ 141.0), OMe-5' (δ 3.87)/C-5' (δ 153.4), and OMe-8'' (δ 3.90)/C-8'' (δ 141.0), and NOESY correlations (Figure 3) between OMe-5' (δ 3.87) and H-6' (δ 6.64). The ^1^H- and ^13^C-NMR spectra (Table 1 and Table 2) of **1** also showed two diastereomers (*M* and *P* in terms of helicity) as in the case of (*M*),(2*S*),(2''*S*)-,(*P*),(2*S*),(2''*S*)-8,5',8''-trihydroxy-7,3',4',7'',3''',4''',5'''-heptamethoxy-5,5''-biflavan (**4**) [5]. The absolute configurations at C-2 and C-2'' were determined as 2*S*,2''*S* by CD comparison with the analogous biflavan, (*M*),(2*S*),(2''*S*)-, (*P*),(2*S*),(2''*S*)-8,5',8''-trihydroxy-7,3',4',7'',3''',4''',5'''-heptamethoxy-5,5''-biflavan (**4**) [5]. The full assignment of ^1^H- and ^13^C-NMR resonances (Table 1 and Table 2) was supported by ^1^H-^1^H COSY, DEPT, HSQC, NOESY (Figure 3), and HMBC (Figure 3) spectral analyses. According to the above data, the structure of **1** was elucidated as (*M*),(2*S*),(2''*S*)-,(*P*),(2*S*),(2''*S*)-7,8,3',4',5',7'',8'',3''',4''',5'''-decamethoxy-5,5''-biflavan.

**Table 1:** ^1^H-NMR data of **1** and **4** [5].

Position	δ_H_			
	1^a^		4 ** ^b^	
	*M*	*P*	*M*	*P*
2	5.02 dd (9.6, 3.0)	5.03 dd (9.6, 3.0)	4.90 d (9.4)	4.90 d (9.4)
3ax	1.90–2.08 m	1.90–2.08 m	1.70–2.13 m	1.70–2.13 m
3eq	2.09–2.18 m	2.09–2.18 m	1.70–2.13 m	1.70–2.13 m
4ax	2.66 ddd (16.4, 11.0, 6.0)	2.54 ddd (16.4, 11.0, 6.0)	2.25–2.72 m	2.25–2.72 m
4eq	2.23 ddd (16.4, 4.4, 4.4)	2.50 ddd (16.4, 4.4, 4.4)	2.25–2.72 m	2.25–2.72 m
6	6.42 s	6.37 s	6.38 s	6.30 s
2'	6.64 s	6.66 s	6.59 d (2.1)	6.59 d (2.1)
6'	6.64 s	6.66 s	6.61 s	6.61 s
2''	5.02 dd (9.6, 3.0)	5.03 dd (9.6, 3.0)	4.97 d (9.9)	4.97 d (9.9)
3''ax	1.90–2.08 m	1.90–2.08 m	1.70–2.13 m	1.70–2.13 m
3''eq	2.09–2.18 m	2.09–2.18 m	1.70–2.13 m	1.70–2.13 m
4''ax	2.66 ddd (16.4, 11.0, 6.0)	2.54 ddd (16.4, 11.0, 6.0)	2.25–2.72 m	2.25–2.72 m
4''eq	2.23 ddd (16.4, 4.4, 4.4)	2.50 ddd (16.4, 4.4, 4.4)	2.25–2.72 m	2.25–2.72 m
6''	6.42 s	6.37 s	6.38 s	6.32 s
2'''	6.64 s	6.66 s	6.79 s	6.82 s
6'''	6.64 s	6.66 s	6.79 s	6.82 s
OMe-7	3.86 s	3.87 s	3.75 s	3.74 s
OH-8			8.13 s	8.13 s
OMe-8	3.90 s	3.89 s		
OMe-3'	3.87 s	3.88 s	3.77 s	3.79 s
OMe-4'	3.85 s	3.86 s	3.66 s	3.68 s
OH-5'			9.17 s	9.20 s
OMe-5'	3.87 s	3.88 s		
OMe-7''	3.86 s	3.87 s	3.75 s	3.74 s
OH-8''			8.13 s	8.13 s
OMe-8''	3.90 s	3.89 s		
OMe-3'''	3.87 s	3.88 s	3.77 s	3.79 s
OMe-4'''	3.85 s	3.86 s	3.66 s	3.68 s
OMe-5'''	3.87 s	3.88 s	3.81 s	3.79 s

^a^ Recorded in CDCl_3_ at 400 MHz. ^b^ Recorded in DMSO-*d*_6_ at 300 MHz. Values in ppm (δ). *J* (in Hz) in parentheses.

**Table 2:** ^13^C-NMR data of **1** and **4** [5].

Position	δ_C_			
	1 ^a^		4 ^b^	
	*M*	*P*	*M*	*P*
2	78.5	78.6	76.8	76.9
3	30.4	30.1	29.7	29.5
4	23.6	23.0	23.3	22.7
5	130.8	130.4	130.0	130.5
6	109.6	109.2	106.2	105.7
7	146.5	146.5	145.9	145.9
8	141.0	141.0	133.7	133.7
9	147.6	147.4	143.7	143.6
10	113.8	113.2	113.7	114.2
1'	136.9	136.9	137.2	137.2
2'	103.2	103.3	101.6	101.7
3'	153.4	153.4	153.0	153.0
4'	136.8	136.8	135.7	135.8
5'	153.4	153.4	150.3	150.4
6'	103.2	103.3	107.2	107.2
2''	78.5	78.6	77.0	77.1
3''	30.4	30.1	29.7	29.5
4''	23.6	23.0	23.5	22.9
5''	130.8	130.4	130.1	130.6
6''	109.6	109.2	106.4	105.9
7''	146.5	146.5	145.9	145.9
8''	141.0	141.0	133.7	133.7
9''	147.6	147.4	143.6	143.6
10''	113.8	113.2	113.7	114.2
1'''	136.9	136.9	137.4	137.4
2'''	103.2	103.3	103.7	103.8
3'''	153.4	153.4	152.8	152.8
4'''	136.8	136.8	136.9	137.0
5'''	153.4	153.4	152.8	152.8
6'''	103.2	103.3	103.7	103.8
OMe-7	56.3	56.3	56.1	56.1
OMe-8	60.8	60.8		
OMe-3'	56.1	56.1	55.7	55.7
OMe-4'	60.9	60.9	59.9	59.9
OMe-5'	56.1	56.1		
OMe-7''	56.3	56.3	56.1	56.1
OMe-8''	60.8	60.8		
OMe-3'''	56.1	56.1	55.7	55.9
OMe-4'''	60.9	60.9	59.9	60.0
OMe-5'''	56.1	56.1		55.9

^a^ Recorded in CDCl_3_ at 100 MHz. ^b^ Recorded in DMSO-*d*_6_ at 75.6 MHz. Values in ppm (δ).

**Figure 4:**
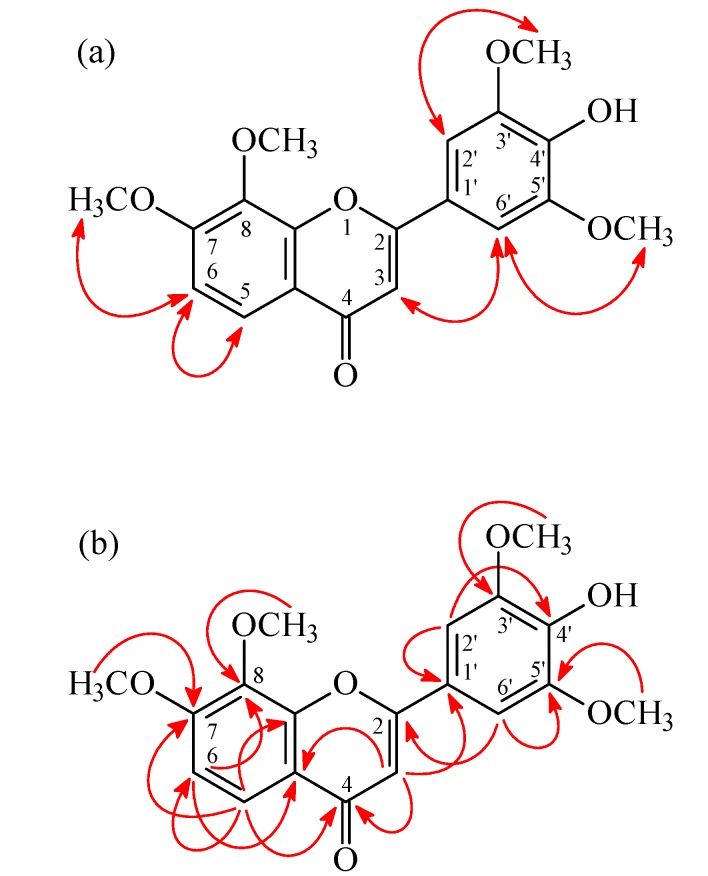
Key NOESY (**a**) and HMBC (**b**) correlations of **2**.

4'-Hydroxy-7,8,3',5'-tetramethoxyflavone (**2**) was isolated as yellowish needles with molecular formula C_19_H_18_O_7_ as determined by positive-ion HR-ESI-MS, showing an [M + Na]^+^ ion at *m*/*z* 381.0952 (calcd for C_19_H_18_O_7_Na, 381.0950). The presence of a carbonyl group was revealed by a band at 1630 cm^−1^ in the IR spectrum, and was confirmed by the resonance at δ 178.0 in the ^13^C-NMR spectrum. The ^1^H-NMR spectrum of **2** showed the presence of four methoxy groups [δ 3.99 (6H, s, OMe-3' and OMe-5'), 4.02 (3H, s, OMe-8), and 4.05 (3H, s, OMe-7)], five aromatic protons [δ 6.72 (1H, s, H-3), 7.22 (2H, s, H-2' and H-6'), and 7.07, 7.96 (each 1H, each d, *J* = 8.8 Hz, H-6 and H-5)], and a hydroxy group [δ 5.88 (1H, br s, D_2_O exchangeable, OH-4')]. The ^1^H- and ^13^C-NMR data of **2** were similar to those of 7,8,3',4',5'-pentamethoxyflavone (**7**) [5], except that the 4'-hydroxy group [δ_H_ 5.88 (1H, br s); δ_C_ 136.5 (C-4')] of **2** replaced the 4'-methoxy group [δ_H_ 3.94 (3H, s); δ_C_ 61.1 (OMe-4'), 141.1 (C-4')] of **7**. This was supported by HMBC correlation observed between H-2'/H-6' (δ 7.22) and C-4' (δ 136.5). On the basis of the evidence above, the structure of **2** was elucidated as 4'-hydroxy-7,8,3',5'-tetramethoxyflavone, which was further substantiated through 2D-experiments, including HSQC, ^1^H-^1^H COSY, HMBC (Figure 4), and NOESY (Figure 4) spectra. This is the first report of the occurrence of **2** in a natural source, although it has been synthesized by Bellini and Venturella [12].

(*R*)-2',β-Dihydroxy-3',4'-dimethoxydihydrochalcone (**3**) was isolated as amorphous powder. The ESI-MS of **3** afforded an [M + Na]^+^ ion at *m*/*z* 325, implying a molecular formula of C_17_H_18_O_5_, which was confirmed by HR-ESI-MS (*m*/*z* 325.1053 [M + Na]^+^, calcd for C_17_H_18_O_5_Na, 325.1052). The IR spectrum showed the presence of OH (3412 cm^−1^) and carbonyl (1625 cm^−1^) groups. Comparison of the ^1^H-NMR data of **3** with those of 3'-methoxy-2',4',β-trihydroxydihydrochalcone (**3a**) [4] suggested that their structures were closely related, except that 4'-methoxy group [δ 3.91 (3H, s)] of **3** replaced the 5'-hydroxy group [δ 6.41 (1H, br s)] of 3'-methoxy-2',4',β-trihydroxydihydrochalcone. This was supported by the NOESY correlations between OMe-4' (δ 3.91) and H-5' (δ 6.46) and by the HMBC correlations between OMe-4' (δ 3.91) and C-4' (δ 158.5). Furthermore, the absolute configuration of **3** was proposed to be *R* by comparing specific rotation data (${\left[\alpha \right]}_{D}^{25}$ +60.5) of **3** with that reported for (*R*)-β-hydroxydihydrochalcone (${\left[\alpha \right]}_{D}^{27}$ +60.8) [13]. By the aid of DEPT, HSQC, HMBC (Table 3), ^1^H-^1^H COSY, and NOESY (Table 3) techniques, and the full ^1^H- and ^13^C-NMR signals of **3** were unambiguously assigned. Therefore, the structure of **3** was determined as (*R*)-2',β-dihydroxy-3',4'-dimethoxydihydrochalcone.

The known isolates were readily identified by a comparison of physical and spectroscopic data (UV, IR, ^1^H-NMR, [α]_D_, and MS) with corresponding authentic samples or literature values, and this included a biflavan, (*M*),(2*S*),(2''*S*)-,(*P*),(2*S*),(2''*S*)-8,5',8''-trihydroxy-7,3',4',7'',3''',4''',5'''-heptame-thoxy-5,5''-biflavan (**4**) [5], three flavones, 5-hydroxy-7-methoxyflavone (**5**) [14], quercetin (**6**) [15], and 7,8,3',4',5'-pentamethoxyflavone (**7**) [5], two flavans, (2*S*)-8,5'-dihydroxy-7,3',4'-trimethoxyflavan (**8**) [5] and (2*S*)-5'-hydroxy-7,8,3',4'-tetramethoxyflavan (**9**) [5], two flavanones, (2*S*)-7-hydroxy-flavanone (**10**) [16] and (2*S*)-7-hydroxy-8-methoxyflavanone (**11**) [4], two benzenoids, (*E*)-ferulic acid (**12**) [17] and gallic acid (**13**) [18], and two steroids, β-sitostenone (**14**) [19] and β-sitosterol (**15**) [20].

**Table 3:** ^1^H-NMR data of **3** and **3a** [4].

Position	3 ^a^			3a ^a,b^
	δ_H_ *J* (Hz)	NOESY	HMBC	δ_H_ *J* (Hz)
2	7.44 br d (7.6)	3, α, β	4, 6, β	7.44 br d (7.6)
3	7.39 br t (7.6)	2, 4	1, 5	7.39 br t (7.6)
4	7.31 br t (7.6)	3, 5	2, 3, 5, 6	7.31 br t (7.6)
5	7.39 br t (7.6)	4, 6	1, 3	7.39 br t (7.6)
6	7.44 br d (7.6)	5, α, β	2, 4, β	7.44 br d (7.6)
α	3.29 dd (17.2, 3.2)	6, β	1, 1', C=O	3.29 dd (17.4, 3.2)
	3.36 dd (17.2, 8.8)	β, 6'	1, 1', β	3.36 dd (17.4, 8.8)
β	5.33 dd (8.8, 3.2)	2, 6, α	1, 2, 6, α, C=O	5.34 dd (8.8, 3.2)
5'	6.46 d (9.0)	6', OMe-4'	1', 3', 4'	6.50 d (9.0)
6'	7.47 d (9.0)	α, 5'	2', 4', C=O	7.40 d (9.0)
OH-β	3.64 br s			3.57 br s
OH-2'	12.62 s		1', 2', 3'	12.67 s
OH-4'				6.41 br s
OMe-3'	3.87 s		3'	4.00 s
OMe-4'	3.91 s	5'	4'	

^a^ Recorded in CDCl_3_ at 400 MHz. Values in ppm (δ). *J* (in Hz) in parentheses. ^b^ **3a** = 3′-methoxy-2',4',β-trihydroxydihydrochalcone [4].

Reactive oxygen species (ROS) (e.g., superoxide anion (O_2_^•−^), hydrogen peroxide) and granule proteases (e.g., elastase, cathepsin G) produced by human neutrophils contribute to the pathogenesis of inflammatory diseases. Inhibition of neutrophil O_2_^•−^ generation by drugs has been proposed as a way to ameliorate inflammatory diseases. The anti-inflammatory effects of the isolated compounds from the stem wood of *M. calabura* were evaluated by suppressing fMet-Leu-Phe (fMLP)-induced O_2_^•−^ generation by human neutrophils. The anti-inflammatory activity data are shown in Table 4. Ibuprofen, a clinically used anti-inflammatory agent, was used as the positive control. From Table 4, six conclusions can be drawn: (a) 4'-Hydroxy-7,8,3',5'-tetramethoxyflavone (**2**), (*M*),(2*S*),(2''*S*)-, (*P*),(2*S*),(2''*S*)-8,5',8''-trihydroxy-7,3',4',7'',3''',4''',5'''-heptamethoxy-5,5''-biflavan (**4**), 5-hydroxy-7-methoxyflavone (**5**), quercetin (**6**), (2*S*)-8,5'-dihydroxy-7,3',4'-trimethoxyflavan (**8**), (2*S*)-7-hydroxy-flavanone (**10**), and (*E*)-ferulic acid (**12**) exhibited inhibition (IC_50_ ≤ 58.4 μM) of superoxide anion generation by human neutrophils in response to formyl-L-methionyl-L-leucyl-L-phenylalanine (fMLP). (b) Biflavan **4** (with 8,5',8''-trihydroxy-7,3',4',7'',3''',4''',5'''-heptamethoxy groups) exhibited more effective inhibition than its analogue **1** (with 7,8,3',4',5',7'',8'',3''',4''',5'''-decamethoxy groups) against fMLP-induced O_2_^•−^ generation. (c) Flavones **5** (with 5-hydroxy-7-methoxy groups) and **6** (with 3,5,7,3',4'-pentahydroxy groups) showed strong inhibition against fMLP-induced superoxide production, but its analogue **7** (with 7,8,3',4',5'-pentamethoxy groups) was inactive. (d) Flavan **8** (with 8,5'-dihydroxy-7,3',4'-trimethoxy groups) exhibited more effective inhibition than its analogue **9** (with 5'-hydroxy-7,8,3',4'-tetramethoxy groups) against fMLP-induced O_2_^•−^ generation. (e) Flavanone **10** (with 7-hydroxy group) exhibited more effective inhibition than its analogue **11** (with 5'-hydroxy-7,3',4'-trimethoxy groups) against fMLP-induced O_2_^•−^ generation. (f) 5-Hydroxy-7-methoxyflavone (**5**), quercetin (**6**), and (2*S*)-7-hydroxyflavanone (**10**) are the most effective among the isolated compounds, with IC_50_values of 1.77 ± 0.70, 3.82 ± 0.46, and 4.92 ± 1.71 μM, respectively, against fMLP-induced superoxide anion generation.

**Table 4:** Inhibitory effects of compounds **1**–**15** from the stem wood of *M. calabura* on superoxide radical anion generation by human neutrophils in response to fMet-Leu-Phe ^a^.

Compounds	IC_50_ (μM) ^a^
(*M*),(2*S*),(2''*S*)-,(*P*),(2*S*),(2''*S*)-7,8,3',4',5',7'',8'',3''',4''',5'''-Decamethoxy-5,5''-biflavan (**1**)	>100
4'-Hydroxy-7,8,3',5'-tetramethoxyflavone (**2**)	58.4 ± 6.2
(*R*)-2',β-Dihydroxy-3',4'-dimethoxydihydrochalcone (**3**)	>100
(*M*),(2*S*),(2''*S*)-,(*P*),(2*S*),(2''*S*)-8,5',8''-Trihydroxy-7,3',4',7'',3''',4''',5'''-heptamethoxy-5,5''-biflavan (**4**)	54.2 ± 5.3
5-Hydroxy-7-methoxyflavone (**5**)	1.77 ± 0.70
Quercetin (**6**)	3.82 ± 0.46
7,8,3',4',5'-Pentamethoxyflavone (**7**)	>100
(2 *S*)-8,5'-Dihydroxy-7,3',4'-trimethoxyflavan (**8**)	56.6 ± 6.2
(2 *S*)-5'-Hydroxy-7,8,3',4'-tetramethoxyflavan (**9**)	>100
(2 *S*)-7-Hydroxyflavanone (**10**)	4.92 ± 1.71
(2 *S*)-5'-Hydroxy-7,3',4'-trimethoxyflavanone (**11**)	>100
(*E*)-Ferulic acid (**12**)	24.18 ± 1.54
Gallic acid (**13**)	>100
β-Sitostenone (**14**)	>100
β-Sitosterol (**15**)	>100
Ibuprofen ^b^	27.5 ± 3.2

^a^ The IC_50_ values were calculated from the slope of the dose-response curves (SigmaPlot). Values are expressed as average ± SEM (*n* = 4); ^b^ Ibuprofen was used as a positive control.

### 2.2. Discussion

A new biflavan **1**, a new flavone **2**, a new dihydrochalcone **3**, and twelve known compounds **4**–**15** were isolated from the stem wood of *M. calabura*. The structures of new compounds **1**–**3** were determined by NMR and MS analyses. Among the known isolates, compound **12** has been found for the first time in this plant species. More discovery of new compounds from the genus *Muntingia* may not only provide more structure-activity data of these isolates, but also contribute to enhancing our understanding of the taxonomy and evolution of the genus *Muntingia*.

Human neutrophils are known to play a significant role in the host defense against microorganisms and in the pathogenesis of various diseases such as ischemia-reperfusion injury, asthma, rheumatoid arthritis, and chronic obstructive pulmonary disease [21,22]. In response to different stimuli, activated neutrophils secrete a series of cytotoxins, such as superoxide anion (O_2_^•−^), a precursor of other reactive oxygen species (ROS), and bioactive lipids [21,23,24]. Suppression of the extensive or inappropriate activation of neutrophils by drugs has been proposed as a way to ameliorate inflammatory diseases. Based on the results of our biological tests (Table 4), 5-hydroxy-7-methoxyflavone (**5**), quercetin (**6**), and (2*S*)-7-hydroxyflavanone (**10**) exhibited potent inhibition with IC_50_ values of 1.77 ± 0.70, 3.82 ± 0.46, and 4.92 ± 1.71 μM, respectively, against fMLP-induced superoxide anion generation. These findings indicated that the promising inhibitory activity against fMLP-induced O_2_^•−^ generation of *M. calabura* and its isolates (especially **5**, **6**, and **10**) could stimulate future development of new anti-inflammatory agents.

## 3. Experimental Section

### 3.1. General Procedures

Melting points were determined on a Yanaco micro-melting point apparatus and were uncorrected. Optical rotations were measured using a Jasco DIP-370 polarimeter in CHCl_3_. Ultraviolet (UV) spectra were obtained on a Jasco UV-240 spectrophotometer. Circular dichroism (CD) spectra were recorded on a Jasco J-810 spectropolarimeter. Infrared (IR) spectra (neat or KBr) were recorded on a Perkin Elmer 2000 FT-IR spectrometer. Nuclear magnetic resonance (NMR) spectra, including correlation spectroscopy (COSY), nuclear Overhauser effect spectrometry (NOESY), heteronuclear multiple-bond correlation (HMBC), and heteronuclear single-quantum coherence (HSQC) experiments, were acquired using a Varian Unity 400 or a Varian Inova 500 spectrometer operating at 400 and 500 MHz (^1^H) and 100 and 125 MHz (^13^C), respectively, with chemical shifts given in ppm (δ) using tetramethylsilane (TMS) as an internal standard. Electrospray ionisation (ESI) and high-resolution electrospray ionization (HRESI)-mass spectra were recorded on a Bruker APEX II or a VG Platform Electrospray ESI/MS mass spectrometer. Silica gel (70–230, 230–400 mesh, Merck) was used for column chromatography (CC). Silica gel 60 F-254 (Merck, Darmstadt, Germany) was used for thin-layer chromatography (TLC) and preparative thin-layer chromatography (PTLC).

### 3.2. Plant Material

The stem wood of *M. calabura* was collected from Changjhih Township, Pingtung County, Taiwan, in August 2011 and identified by Dr. J. J. Chen. A voucher specimen (MC-201108) was deposited in the Department of Pharmacy, Tajen University, Pingtung, Taiwan.

### 3.3. Extraction and Isolation

The dried stem wood (3.7 kg) of *M. calabura* was pulverized and extracted three times with MeOH (30 L each) for 3 days. The MeOH extracts were concentrated under reduced pressure at 35 °C, and the residue (360 g) was partitioned between CH_2_Cl_2_ and H_2_O (1:1). The CH_2_Cl_2_ layer was concentrated to give a residue (fraction A, 105 g). The water layer was further extracted with *n*-BuOH, and the *n*-BuOH-soluble part (fraction B, 121 g) and the water-solubles (fraction C, 134 g) were separated. Fraction A (105 g) was chromatographed on silica gel (70–230 mesh, 5.1 kg), eluting with CH_2_Cl_2_, gradually increasing the polarity with MeOH to give 11 fractions: A1 (3 L, CH_2_Cl_2_), A2 (3.5 L, CH_2_Cl_2_/MeOH, 98:1), A3 (3 L, CH_2_Cl_2_/MeOH, 95:1), A4 (3 L, CH_2_Cl_2_/MeOH, 90:1), A5 (4 L, CH_2_Cl_2_/MeOH, 80:1), A6 (3 L, CH_2_Cl_2_/MeOH, 70:1), A7 (4 L, CH_2_Cl_2_/MeOH, 50:1), A8 (4 L, CH_2_Cl_2_/MeOH, 10:1), A9 (3 L, CH_2_Cl_2_/MeOH, 5:1), A10 (4 L, CH_2_Cl_2_/MeOH, 1:1), A11 (3.5 L, MeOH). Fraction A2 (9.4 g) was washed with MeOH and filtered to yield **14** (71 mg) after recrystallization (MeOH). The filtrate was chromatographed on silica gel (230–400 mesh) eluting with *n*-hexane/EtOAc (15:1–0:1) to give 10 fractions (each 800 mL, A2-1–A2-10). Fraction A2-3 (102 mg) was purified by preparative TLC (silica gel, *n*-hexane/acetone, 10:1) to obtain **15** (7.2 mg). Fraction A3 (8.8 g) was chromatographed further on silica gel (230–400 mesh, 398 g) eluting with CH_2_Cl_2_/MeOH (15:1–0:1) to give 9 fractions (each 850 mL, A3-1–A3-9). Fraction A3-3 (95 mg) was purified by preparative TLC (silica gel, CHCl_3_/acetone, 15:1) to obtain **5** (4.7 mg). Fraction A3-5 (104 mg) was purified by preparative TLC (silica gel, *n*-hexane/acetone, 2:1) to afford **9** (3.8 mg). Fraction A3-8 (102 mg) was purified by preparative TLC (silica gel, *n*-hexane/acetone, 1:1) to afford **11** (4.6 mg). Fraction A4 (10.1 g) was chromatographed further on silica gel (230–400 mesh, 462 g) eluting with CHCl_3_/MeOH (10:1–0:1) to give 12 fractions (each 750 mL, A4-1–A4-12). Fr. A4-2 (113 mg) was further purified by preparative TLC (silica gel, *n*-hexane/acetone, 5:1) to obtain **1** (3.5 mg). Fraction A4-3 (364 mg) was purified by MPLC (16.7 g of SiO_2_, 230–400 mesh, *n*-hexane/EtOAc 15:1–0:1, 180-mL fractions) to give 8 subfractions: Frs. A4-3-1–A4-3-8. Fr. A4-3-3 (48 mg) was further purified by preparative TLC (silica gel, *n*-hexane/acetone, 3:1) to afford **4** (4.2 mg). Fraction A4-4 (136 mg) was purified further by preparative TLC (silica gel, CH_2_Cl_2_/acetone, 4:1) to obtain **7** (5.3 mg). Fraction A4-5 (145 mg) was purified further by preparative TLC (silica gel, CHCl_3_/acetone, 4:1) to give **2** (3.6 mg). Fraction A4-6 (133 mg) was purified further by preparative TLC (silica gel, CHCl_3_/acetone, 5:1) to give **12** (5.7 mg). Fraction A5 (9.2 g) was chromatographed further on silica gel (230–400 mesh, 437 g) eluting with CHCl_3_/MeOH (10:1–0:1) to give 10 fractions (each 750 mL, A5-1–A5-10). Fraction A5-2 (132 mg) was further purified by preparative TLC (*n*-hexane/acetone, 4:3) to obtain **3** (3.7 mg). Fraction A5-3 (124 mg) was further purified by preparative TLC (*n*-hexane/acetone, 3:2) to obtain **10** (4.8 mg). Fraction A5-4 (116 mg) was purified further by preparative TLC (silica gel, *n*-hexane/EtOAc, 1:1) to afford **8** (4.1 mg). Fraction A8 (8.4 g) was chromatographed further on silica gel (230–400 mesh, 383 g) eluting with CH_2_Cl_2_/MeOH (6:1–0:1) to give 8 fractions (each 800 mL, A8-1–A8-8). Fraction A8-1 (108 mg) was purified by preparative TLC (silica gel, EtOAc/MeOH, 1:1) to afford **13** (4.4 mg). Fraction A8-4 (122 mg) was purified by preparative TLC (silica gel, CH_2_Cl_2_/MeOH, 2:1) to afford **6** (4.5 mg).

*(M),(2S),(2''S)-,(P),(2S),(2''S)-7,8,3',4',5',7'',8'',3''',4''',5'''-Decamethoxy-5,5''-biflavan* (**1**). Colorless prisms (CH_2_Cl_2_/MeOH), m.p. > 225 °C (dec). ${\left[\alpha \right]}_{D}^{25}$: +12.8 (*c* 0.16, CHCl_3_). UV (MeOH): λ_max_ (log ε) = 246 (4.21), 268 (3.86) nm. CD (MeOH, Δε): 275 (−0.4), 289 (−2.0), 304 (0) nm. IR (KBr): υ_max_ = 1602, 1515, 1452 (aromatic ring C=C stretch) cm^−1^. ^1^H-NMR spectroscopic data, see Table 1. ^13^C-NMR spectroscopic data, see Table 2. ESI-MS: *m*/*z* = 741 [M + Na]^+^. HR-ESI-MS: *m*/*z* = 741.2889 [M + Na]^+^ (calcd for C_40_H_46_O_12_Na: 741.2887).

*4'-Hydroxy-7,8,3',5'-tetramethoxyflavone* (**2**). Yellowish needles (MeOH); m.p. 221–223 °C. UV (MeOH): λ_max_ (log ε) = 242 (4.23), 269 (4.29), 312 (4.28) nm. IR (KBr): υ_max_ 3290 (OH), 1630 (C=O) cm^−1^. ^1^H-NMR (CDCl_3_, 500 MHz): δ = 3.99 (6H, s, OMe-3' and OMe-5'), 4.02 (OMe-8), 4.05 (OMe-7), 6.72 (H-3), 7.07 (1H, d, *J* = 8.8 Hz, H-6), 7.22 (2H, s, H-2' and H-6'), 7.96 (1H, d, *J* = 8.8 Hz, H-5). ^13^C-NMR (CDCl_3_, 125 MHz): δ = 56.3 (OMe-3'), 56.3 (OMe-5'), 56.4 (OMe-7), 61.4 (OMe-8), 104.1 (C-2'), 104.1 (C-6'), 106.4 (C-3), 110.0 (C-6), 118.5 (C-10), 121.0 (C-5), 126.9 (C-1'), 136.5 (C-4'), 136.8 (C-8), 147.8 (C-3'), 147.8 (C-5'), 150.4 (C-9), 156.6 (C-7), 162.7 (C-2), 178.0 (C-4). ESI-MS: *m*/*z* = 381 [M + Na]^+^. HR-ESI-MS: *m*/*z* = 381.0952 [M + Na]^+^ (calcd for C_1__9_H_18_O_7_Na: 381.0950).

*(R)-2',β-Dihydroxy-3',4'-dimethoxydihydrochalcone* (**3**). Amorphous powder. ${\left[\alpha \right]}_{D}^{25}$: +60.5 (*c* 0.16, CHCl_3_). UV (MeOH): λ_max_ (log ε) = 211 (4.05), 230 (sh, 3.75), 287 (3.85) nm. IR (KBr): υ_max_ = 3412 (OH), 1625 (C=O) cm^−1^. ^1^H-NMR spectroscopic data, see Table 3. ^13^C-NMR (CDCl_3_, 100 MHz): δ = 52.6 (C-α), 70.2 (C-β), 56.1 (OMe-4'), 60.6 (OMe-3'), 102.9 (C-5'), 114.1 (C-1'), 125.9 (C-4), 126.0 (C-6'), 127.8 (C-2 and C-6), 128.7 (C-3 and C-5), 136.3 (C-3'), 143.2 (C-1), 156.9 (C-2'), 158.5 (C-4'), 200.6 (C=O). ESI-MS: *m*/*z* = 325 [M + Na]^+^. HR-ESI-MS: *m*/*z* = 325.1053 [M + Na]^+^ (calcd for C_1__7_H_1__8_O_5_Na: 325.1052).

### 3.4. Biological Assay

The effect of the isolated compounds on neutrophil pro-inflammatory response was evaluated by monitoring the inhibition of superoxide anion generation in fMLP-activated human neutrophils in a concentration-dependent manner. The purity of the tested compounds was >98% as identified by NMR and MS.

#### 3.4.1. Preparation of Human Neutrophils

Human neutrophils from venous blood of healthy, adult volunteers (20–30 years old) obtained by venipuncture were isolated using a standard method of dextran sedimentation prior to centrifugation in a Ficoll Hypaque gradient and hypotonic lysis of erythrocytes [25]. Purified neutrophils containing >98% viable cells, as determined by the trypan blue exclusion method [26], were re-suspended in a calcium (Ca^2+^)-free HBSS buffer at pH 7.4 and were maintained at 4 °C prior to use. The protocol was approved by the Institutional Review Board at Chang Gung Memorial Hospital. All donors gave written consent. The Medical Ethics Committee of Chang Gung Memorial Hospital approved this consent procedure.

#### 3.4.2. Measurement of Superoxide Anion Generation

The assay for measurement of O_2_^•−^ generation was based on the SOD-inhibitable reduction of ferricytochrome *c* [27]. In brief, neutrophils (1 × 10^6^ cells/mL) pretreated with the various test agents at 37 °C for 5 min were stimulated with fMLP (1 μmol/L) in the presence of ferricytochrome *c* (0.5 mg/mL). Extracellular O_2_^•−^ production was assessed with a UV spectrophotometer at 550 nm (Hitachi U-3010, Tokyo, Japan). The percentage of superoxide inhibition of the test compound was calculated as the percentage of inhibition = {(control − resting) − (compound − resting)}/(control − resting) × 100. The software, SigmaPlot was used for determining the IC_50_ values.

#### 3.4.3. Statistical Analysis

Results were expressed as the mean ± SEM, and comparisons were made using Student’s *t*-test. A probability of 0.05 or less was considered significant. The software SigmaPlot was used for the statistical analysis.

## 4. Conclusions

Fifteen compounds, including three new compounds **1**–**3**, were isolated from the stem wood of *M. calabura*. The structures of these compounds were established on the basis of spectroscopic data. Reactive oxygen species (ROS) (e.g., superoxide anion (O_2_^•−^), hydrogen peroxide) produced by human neutrophils contribute to the pathogenesis of inflammatory diseases. The effects on neutrophil pro-inflammatory responses of isolates were evaluated by suppressing fMLP/CB-induced O_2_^•−^ generation by human neutrophils. The results of anti-inflammatory experiments indicate that compounds **2**, **4**–**6**, **8**, **10**, and **12** can significantly inhibit fMLP-induced O_2_^•−^ generation. 5-Hydroxy-7-methoxyflavone (**5**), quercetin (**6**), and (2*S*)-7-hydroxyflavanone (**10**) are the most effective among the isolated compounds, with IC_50_values of 1.77 ± 0.70, 3.82 ± 0.46, and 4.92 ± 1.71 μM, respectively, against fMLP-induced superoxide anion generation. Our study suggests *M. calabura* and its isolates (especially **5**, **6**, and **10**) could be further developed as potential candidates for the treatment or prevention of various inflammatory diseases.

## Supplementary Materials

Supplementary materials can be accessed at: http://www.mdpi.com/1420-3049/19/12/20521/s1.
