# Supplementary material for: Biflavans, Flavonoids, and a Dihydrochalcone from the Stem Wood of Muntingia calabura and Their Inhibitory Activities on Neutrophil Pro-Inflammatory Responses
Source: Molecules. 2014 Dec 8;19(12):20521–35. doi: 10.3390/molecules191220521 (PMC6271443; doi:10.3390/molecules191220521)

Supplementary Materials

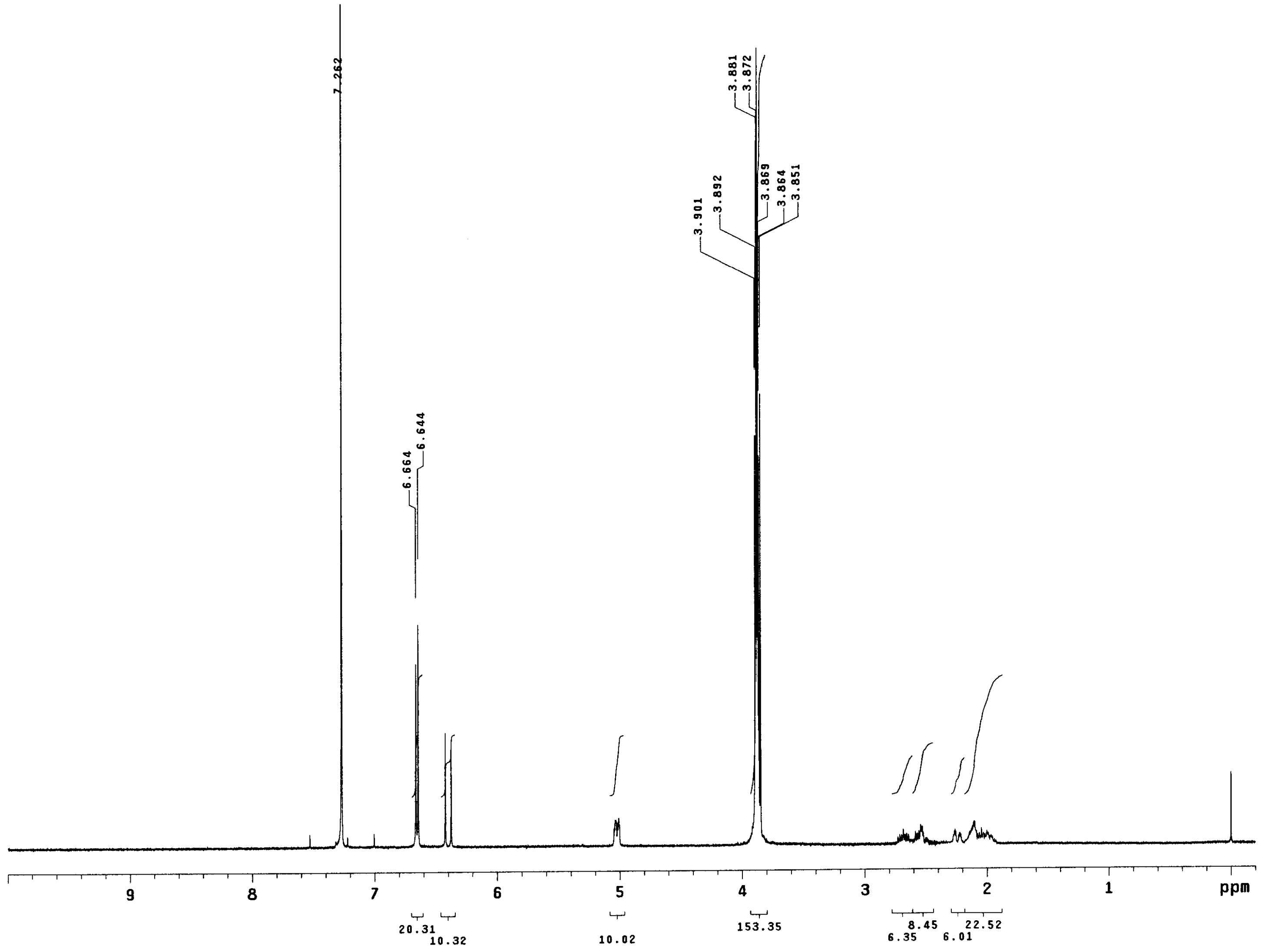

Figure S1. ^{1}H-NMR spectrum of **1** ($CDCl_3$, 400 MHz).

Figure S2. ^{13}C-NMR spectrum of **1** ($CDCl_3$, 100 MHz).

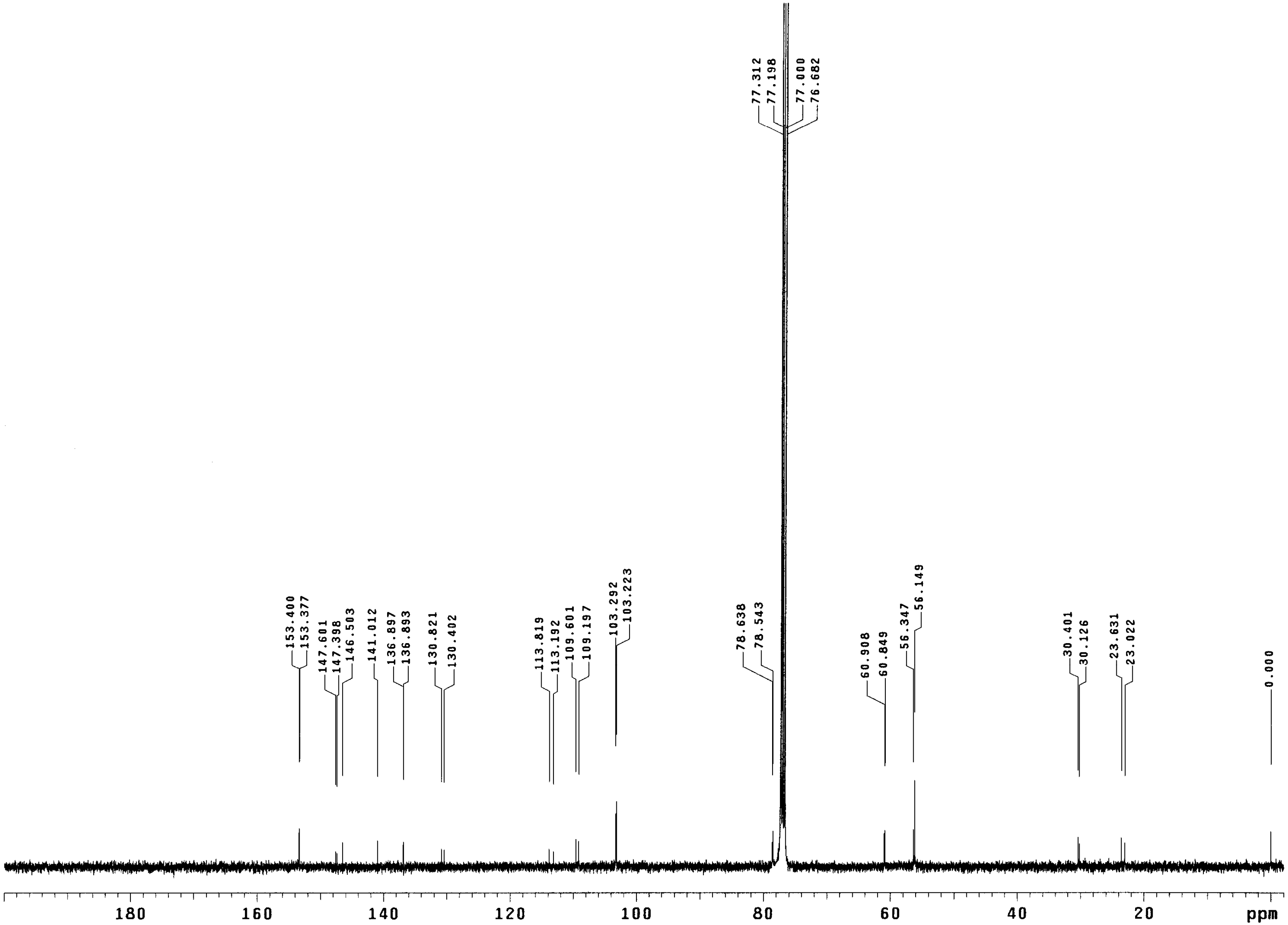

Figure S3. ^{1}H-NMR spectrum of 2 ($CDCl_3$, 500 MHz).

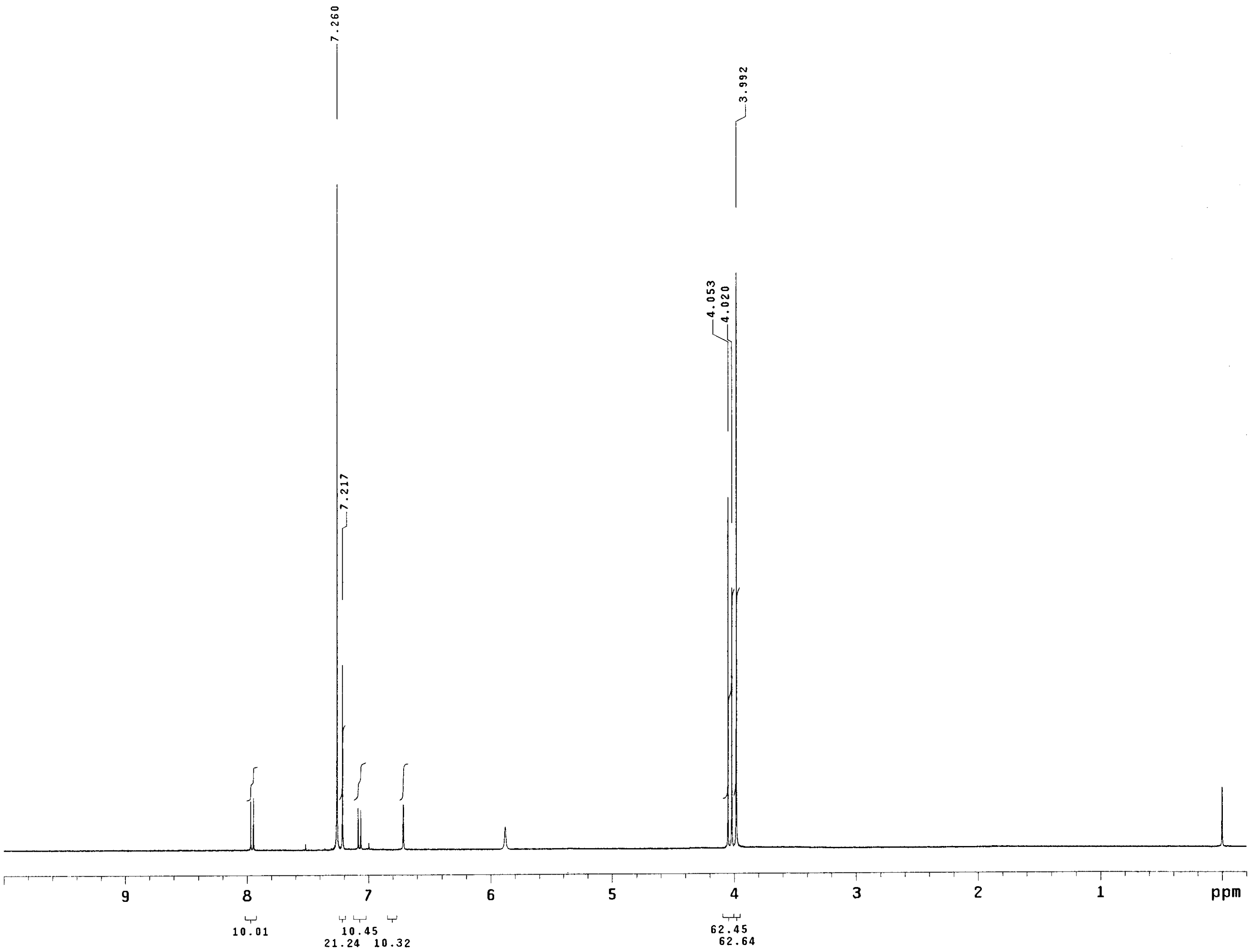

Figure S4. ^{13}C-NMR spectrum of **2** ($CDCl_3$, 125 MHz).

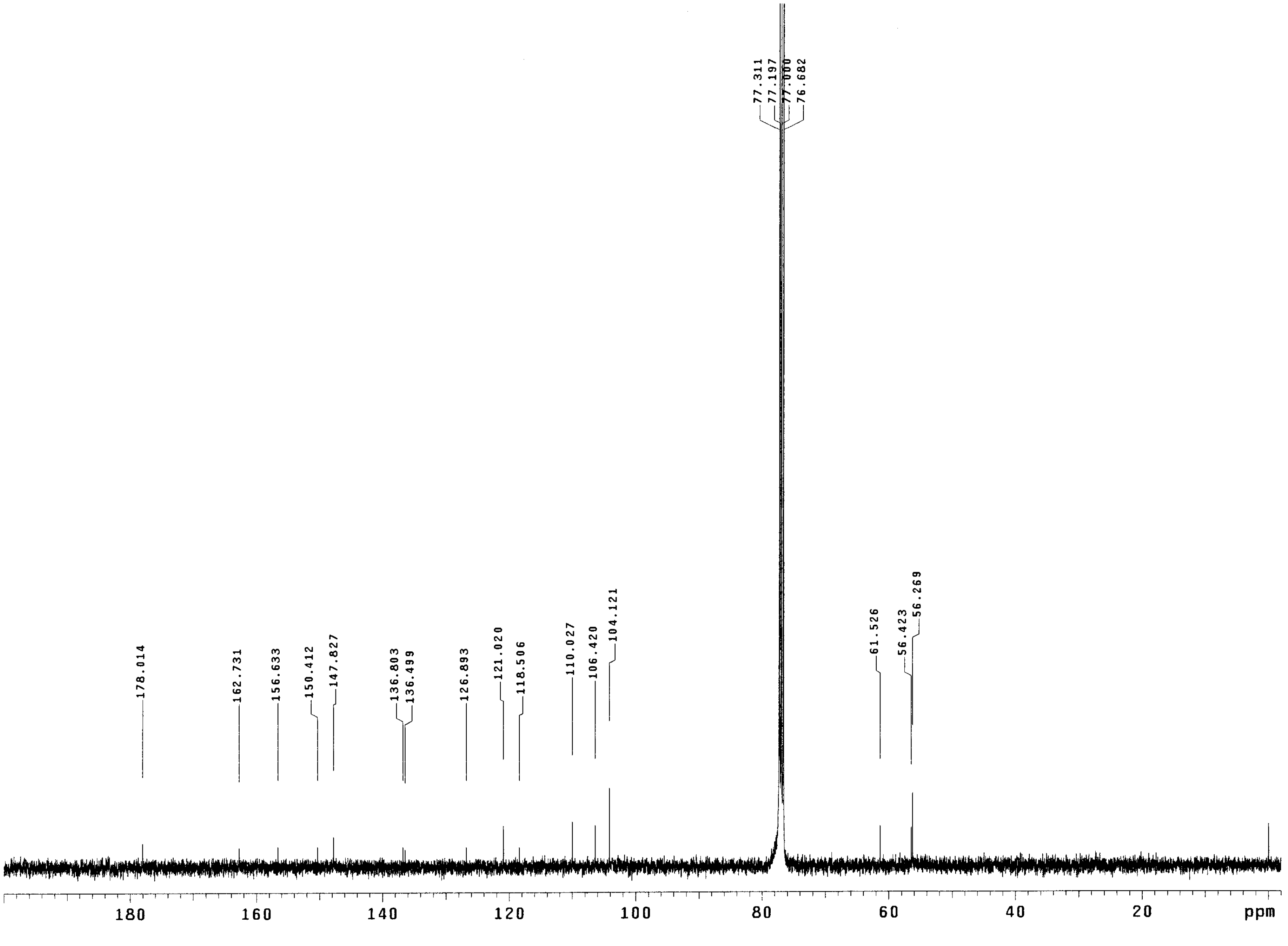

Figure S5. ^{1}H-NMR spectrum of **3** ($CDCl_3$, 400 MHz).

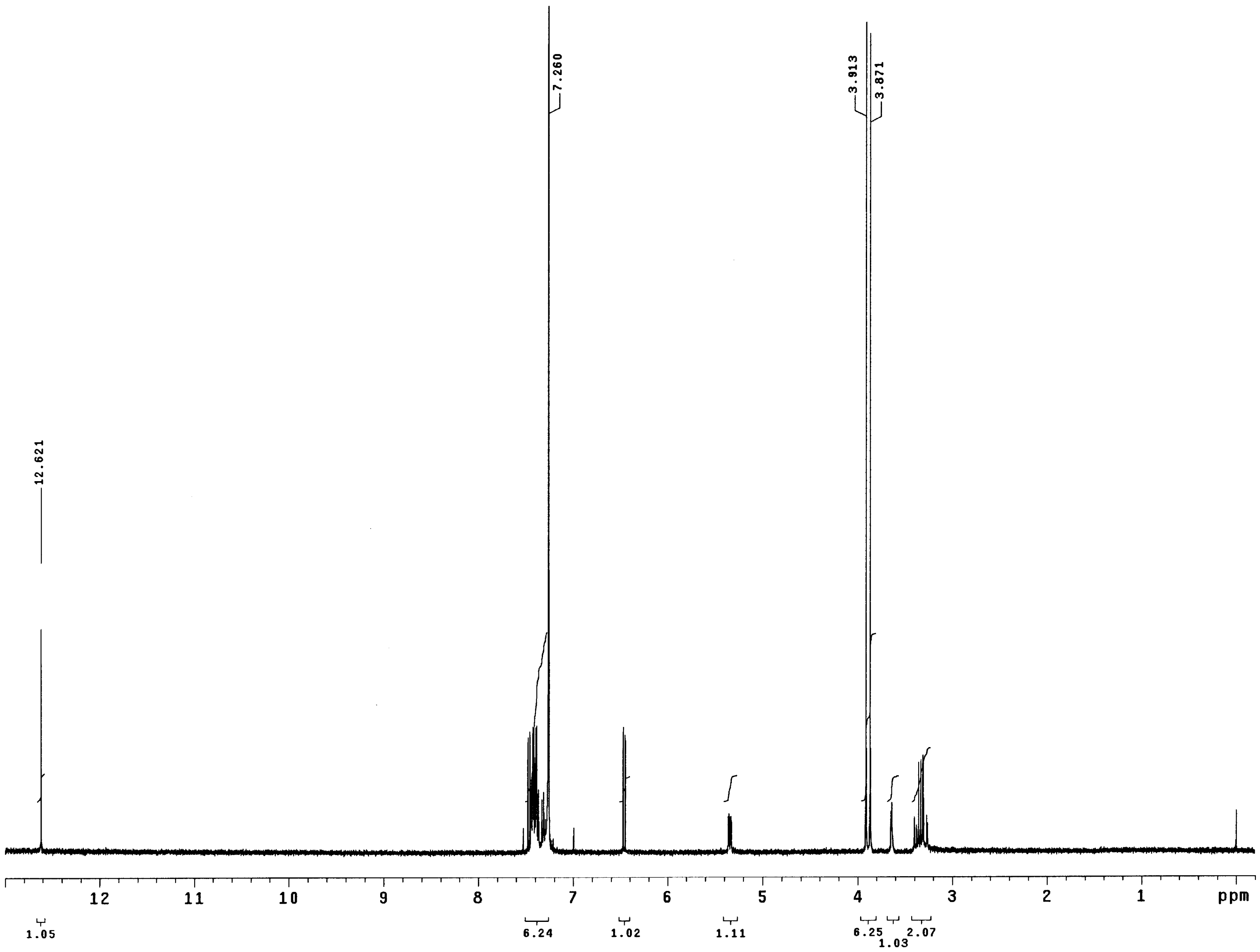

Figure S6. ^{13}C-NMR spectrum of **3** ($CDCl_3$, 100 MHz).

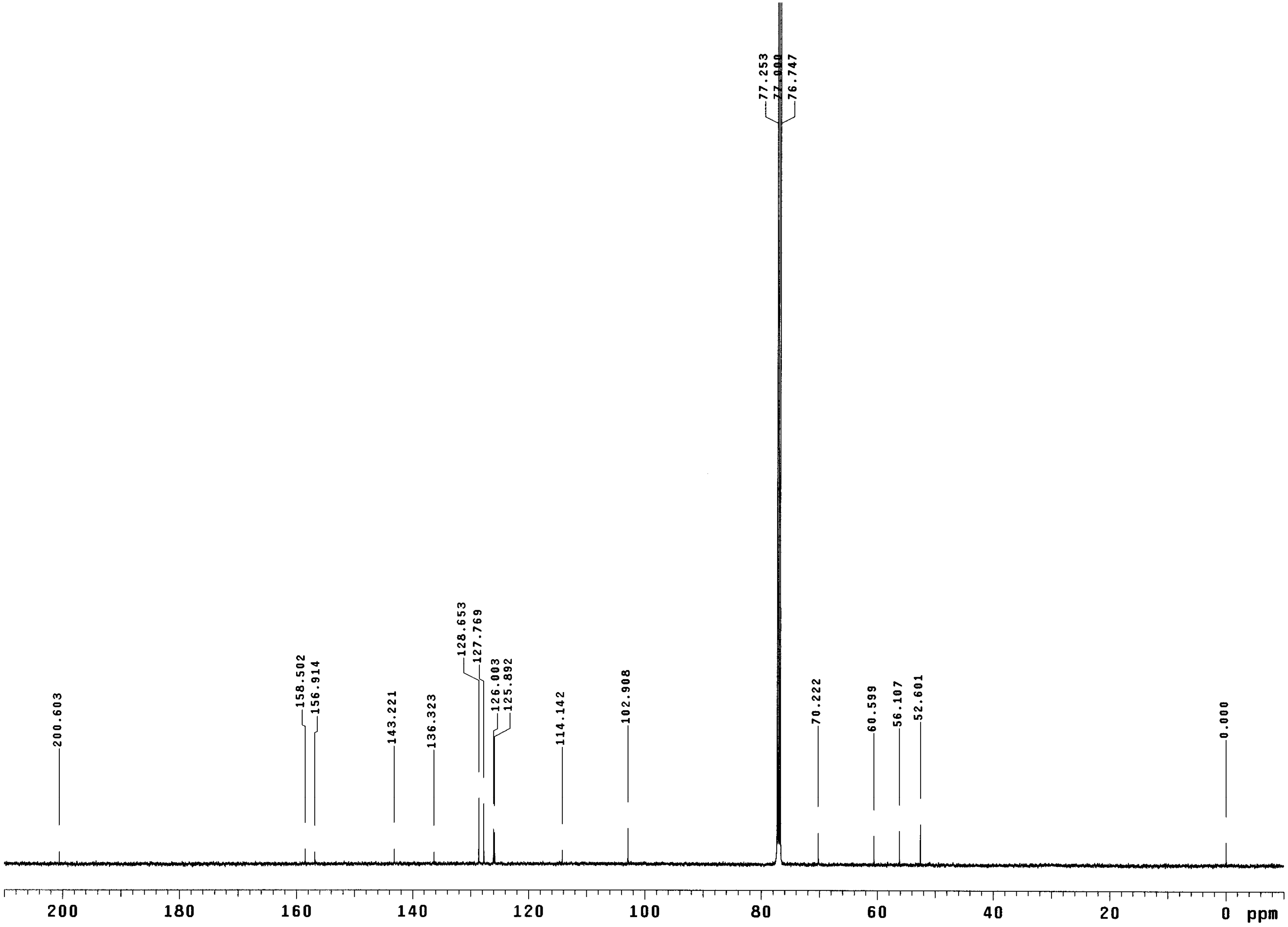

Supplement: Supplementary file 1 [file molecules-19-20521-s001.pdf]
